# Duration of physical activity, sitting, sleep and the risk of total knee replacement among Chinese in Singapore, the Singapore Chinese Health Study

**DOI:** 10.1371/journal.pone.0202554

**Published:** 2018-09-04

**Authors:** Ying Ying Leung, Hamid Rahmatullah Bin Abd Razak, Mohammad Talaei, Li-Wei Ang, Jian-Min Yuan, Woon-Puay Koh

**Affiliations:** 1 Health Services and Systems Research, Duke-NUS Medical School, Singapore, Singapore; 2 Department of Rheumatology and Immunology, Singapore General Hospital, Singapore, Singapore; 3 Department of Orthopaedic Surgery, Singapore General Hospital, Singapore, Singapore; 4 Epidemiology & Disease Control Division, Ministry of Health, Singapore, Singapore; 5 Division of Cancer Control and Population Sciences, UPMC Hillman Cancer Center, University of Pittsburgh, Pittsburgh, Pennsylvania, United States of America; 6 Department of Epidemiology, Graduate School of Public Health, University of Pittsburgh, Pittsburgh, Pennsylvania, United States of America; 7 Saw Swee Hock School of Public Health, National University of Singapore, Singapore, Singapore; Huazhong University of Science and Technology, CHINA

## Abstract

**Objectives:**

While the effect of physical activity on knee osteoarthritis (KOA) remains controversial, how sitting and sleep durations affect KOA is unknown. We evaluated the association between durations of physical activity, sitting and sleep, and incidence of total knee replacement (TKR) due to severe KOA.

**Methods:**

We used data from the Singapore Chinese Health Study, a prospective cohort of 63,257 Chinese, aged 45–74 years at recruitment from 1993–1998. Height, weight, lifestyle factors, hours of sitting and sleep per day, and hours of moderate activity, strenuous sports or vigorous work per week were assessed through in-person interviews using structured questionnaires. Incident cases of TKR were identified via record linkage with nationwide hospital discharge database.

**Results:**

Compared to those with <0.5 hour/week of moderate physical activity, participants with ≥5 hour/week had increased risk of TKR risk [hazard ratio (HR) 1.16, 95% confidence interval (CI) 1.00–1.35]. Conversely, duration of sitting activities, especially sitting at work, was associated with reduced risk in a stepwise manner. Compared to <4 hour/day of sitting, those with ≥12 hour/day had the lowest risk (HR 0.76, 95% CI 0.60–0.96, *p* for trend = 0.02). Sleep duration was inversely associated with reduced risk of TKR in a dose-dependent manner; compared to those with sleep ≤ 5 hour/day, participants with ≥ 9 hour/day had the lowest risk (HR 0.55, 95% CI) 0.43–0.70, *p* for trend <0.001).

**Conclusion:**

While prolonged sitting or sleeping duration could be associated with reduced risk of severe KOA, extended duration of physical activity could be associated with increased risk.

## Introduction

Knee osteoarthritis (KOA) is among the leading causes of disability, and accounts for an increasing burden among the aged population worldwide [1]. In many studies, including ours [2], besides increasing age, body mass index (BMI), which is a measure of the biochemical effect of body weight loading on the knee joints, is the most important risk factor for KOA. Intuitively, it is logical to perceive any physical activity that increases weight-loading on the knee joints to worsen the progression of KOA. However, there is substantial evidence supporting the benefits of prescribed structured exercise regimen in the clinical management of KOA by strengthening the muscles and stabilizing the knee joint [3]. Hence, it remains unclear whether the level of physical activity among the general population is a risk or protective factor for KOA. Differences in study designs, characteristics of participants, variations in the measurement of intensity, duration, and nature of physical activity, as well as definition of KOA, are other reasons for the conflicting and inconclusive results in the current literature.

Two systematic reviews evaluated the effect of physical activity [4] and running [5] in KOA in 10 and 15 population-based studies, respectively, and both reported inconclusive finding overall due to methodological heterogeneity of the studies. A recent meta-analysis evaluated the association of recreational and competitive running with KOA, and reported that recreational runners had a lower occurrence of KOA compared with competitive runners and sedentary non-running controls, thus implying a U-shape relationship between intensity of running and risk of KOA [6]. Walking activities, measured more accurately through pedometers, have been shown to be associated with knee joint abnormality such as bone marrow oedema lesions, and meniscus and cartilage defects on knee magnetic resonance imaging (MRI), both in a cross-sectional study [7] as well as in a longitudinal study that examined knee structural change over time [8].

Conversely, sitting and sleeping have the reverse effect of weight-loading on the knees, and should therefore logically confer protection on risk or progression of KOA by reducing weight-bearing on the knee joints. However, there are only a few studies that have studied the effect of sitting on progression of KOA, and none on the effect of sleeping. Therefore, we evaluated the associations of physical activity, sitting and sleep with the risk of total knee replacement surgery (TKR) due to severe KOA in a large prospective population study among middle-aged and elderly Chinese in Singapore.

## Methods

### Study population

The Singapore Chinese Health Study is a prospective cohort study of 63,257 Chinese (27,959 men and 35,298 women) aged 45–74 years that were enrolled from 1993 to 1998 in Singapore [9]. Participants were recruited from public housing estates, where 86% of Singapore’s population lived at the time of recruitment. This study was approved by the Institutional Review Boards at the National University of Singapore and the University of Pittsburgh. All methods were carried out in accordance with the Declaration of Helsinki, and all subjects have signed informed consent prior to participation.

### Baseline exposure assessment

Participants were interviewed in-person using a structured questionnaire at recruitment to obtain information on education level, cigarette smoking, alcohol consumption, comorbidities, habitual physical activity, as well as sitting and sleeping hours. For physical activity, participants were asked in the last year, the average number of hours per week spent separately on 1) moderate activity such as brisk walking, bowling, bicycling on level ground, and tai chi or chi kung, 2) vigorous work such as moving heavy furniture, loading or unloading trucks, shoveling, or equivalent manual labor, and 3) strenuous sports such as jogging, bicycling on hills, tennis, squash, swimming laps, or aerobics. There were eight options provided for the response to each group of activities: never, 0.5–1 hour, 2–3 hours, 4–6 hours, 7–10 hours, 11–20 hours, 21–30 hours, and 31 hours and more. The physical activity portion of the questionnaire was modeled after the European Prospective Investigation in Cancer (EPIC) study physical activity questionnaire, which has been shown to have acceptable measurement characteristics for ranking participants according to their level of total physical activity [10]. Participants were also asked about in the last year, the average number of hours per day spent separately for separate sitting activities: “sitting at work”, “watching TV”, “sitting at meals”, and “other sitting activities such as reading, playing cards, sewing, etc.”, and there were seven response categories: never, less than 1 hour, 1–2 hours, 3–4 hours, 5–6 hours, 7–10 hours, and 11 hours and more. Usual sleep duration was assessed by the following question: “On average, during the last year, how many hours in a day did you sleep?” Response categories were ≤5, 6, 7, 8, 9, and ≥10 hours. BMI was computed using the formula of weight in kilograms divided by height in meters squared (kg/m^2^), using self-reported information about body weight and height. A total of 10,349 cohort subjects (16%) did not report either weight and/or height, and their BMI was calculated using imputed weight and/or height obtained from the linear regression equation: weight = y-intercept + gradient X height, where values for the y-intercept and gradient were derived from gender-specific weight-height regression lines obtained from all subjects with known heights and weights. This method of data imputation was described in detail previously [11]. Comorbidities were collected by asking “Have you been told by a doctor that you have hypertension, diabetes, coronary artery disease or stroke? The robustness and accuracy the self-reported diabetes (98.9%) and hypertension (88%) data of in this cohort was previously reported [12–13].

### Identification of incident cases of TKR

We identified participants who underwent TKR due to severe KOA via record linkage with the nationwide MediClaim System hospital discharge database through 31 December 2011. The system has captured surgical procedures in Singapore since 1990 and collects up to three discharge diagnoses per patient in public and private hospitals based on the ninth revision of the International Statistical Classification of Diseases and Related Health Problems (ICD-9). We only included first-time TKR cases for severe KOA (ICD-9 code 715), and excluded other TKR cases due to other diagnoses such as septic arthritis, osteomyelitis, villonodular synovitis, rheumatoid arthritis, psoriatic arthritis, ankylosing spondylitis, and other inflammatory arthritis, or secondary causes of KOA such as avascular or aseptic necrosis of joint, meniscus or ligament injuries, and other congenital or acquired deformities of the knee (n = 89). For this analysis, we also excluded 128 prevalent cases of TKR which occurred prior to subject enrolment into the cohort [14]. We identified death of participants through record linkage with the Singapore Registry of Births and Deaths. As of December 2011, only 47 subjects were known to be lost to follow-up due to migration etc., suggesting the ascertainment of vital status for cohort subjects was virtually complete.

### Statistical analysis

We used Cox proportional hazards regression models to calculate hazard ratios (HRs) and their respective 95% confidence intervals (CIs) for the risk of TKR associated with number of hours per week spent in physical activity (moderate activity, vigorous work, strenuous sports, and total), and number of hours per day spent in various sitting activities (at work, for TV, other sitting activities and total) and sleep. Person-years for each participant were calculated from the date of recruitment until time of death, lost-to-follow-up, TKR, or December 31, 2011, whichever came first. For the analyses on physical activity, from the pre-determined categorical responses given by the participants, we used the same cut-off values to create categories of <0.5 h/week, 0.5-<2 h/week, 2-<5 h/week and ≥ 5 h/week, as these categories were reasonable and provided sufficient statistical power in relation to the distribution of duration for moderate activity, strenuous sports and vigorous work, as well as the sum of these three types of physical activity in this cohort. For sitting activities, we adopted the same approach and used the same duration for the referent groups (<1 h/day), but had to use longer durations to create categories for sitting at work, since the duration for sitting at work could be expected to be longer than the other sitting activities. Similarly, for total sitting duration, we used cut-off values to create categories of <4 h/day, 4-<6 h/day, 6-<8 h/day, 8-<12 h/day and ≥ 12 h/day, as these categories were reasonable and provided sufficient statistical power in analysis, according to the distribution in this cohort.

Proportional hazard assumption was tested using Schoenfeld residuals test, and no violation was observed. Three hierarchical multivariable models were used for data analysis; Model 1 comprised age at recruitment (years), sex, year of recruitment (1993–1995, 1995–1998), dialect group (Hokkien, Cantonese), level of education (no formal education, primary school, secondary school or higher), smoking status (never, former, or current), BMI (kg/m^2^), and self-reported histories of physician-diagnosed type 2 diabetes, hypertension, coronary heart disease and stroke. In Model 2, we added the two other factors of interest to Model 1; for example, physical activity and sitting hours were included as covariates for the association between sleep and TKR. Specific analysis for strenuous sports (hours/week), vigorous work (hours/week) and moderate activity (hours/week) also included adjustment for the other two types of physical activity. Similarly, specific analysis for sitting at work, sitting for TV and other sitting activities also included adjustment for the other two types of sitting activities. In Model 3, we further adjusted for menopausal status (yes, no) and use of hormonal replacement therapy in women (yes, no), weekly use of vitamins/minerals (yes, no), daily energy intake (kcal/day) and quartile scores according to the Alternate Healthy Eating Index (AHEI) as a measure of diet quality. The AHEI 2010 measures dietary intake of seven food groups (vegetables, fruit, whole grains, juices, nuts and legumes, red/process meat and alcohol) and four nutrients (trans-fat, long chain n-3 fatty acids, polyunsaturated fatty acids and sodium) based on the scoring criteria predictive of chronic disease risk in cohorts in the United States [15], and has been shown to correlate with risks of acute myocardial infarction [16] and osteoporosis [17]. *P* values for trend were tested by including ordinal categories for the exposures of interest as continuous variables in the models.

Sensitivity analysis was performed by excluding all participants with less than 5 years of follow-up, including TKR cases that had undergone surgery within the first five years. We also excluded participants with imputed BMI due to missing weight, in order to exclude potential bias in using imputed BMI. Potential interactions with gender or BMI were tested using likelihood ratio test of the cross-product terms between exposure status and gender or BMI (<23 and ≥ 23 kg/m^2^). We used a lower BMI cut-off at ≥ 23 kg/m^2^ as overweight or obese as this has been proposed by World Health Organization to be more appropriate for Asians [18]. All statistical analyses were conducted using SAS Version 9.4 (SAS Institute, Inc., Cary, North Carolina). All reported *p* values were two-sided, and *p* < 0.05 was considered statistically significant.

## Results

In our study population, the mean (±SD) duration spent on all types of physical activity was 1.59 ± 4.18 hours/week, the mean duration on sitting activities was 6.51 ± 3.6 hours/day, and the mean sleeping duration was 7.01 ± 1.1 hours/day. About 67.1% of cohort participants spent less than 0.5 hour on physical activity per week, while 9.4% spent 0.5 hour to <2 hours per week, 10.3% spent 2 hours to <5 hours per week, and 13.2% spent more than 5 hours per week on physical activity. The durations of physical activity, sitting activities and sleep were weakly correlated with one another (Spearman correlation coefficients < 0.08). Age was comparable in extreme categories of physical activity and sleep, but individuals with sitting time ≥ 12 hours/day were substantially younger than those who sat < 4 hours/day (Table 1). Among our participants, women were more likely to sleep less than 5 hours/day, have physical activity of less than 0.5 hours/week, and sit less than 4 hours/day. Individuals with higher education were more likely to have physical activity more than 5 hours/week, and also more likely to sit more than 12 hours/day, compared to those without formal education. BMI was comparable across all extreme groups in this population.

**Table 1:** Participant characteristics according to extreme categories of sleep, strenuous sport, and sitting at work, the Singapore Chinese Health Study.

	Total physical activity		Sitting		Sleep	
Characteristics	< 0.5 h/w	≥ 5 h/w	< 4 h/d	≥ 12 h/d	≤ 5 h/d	≥ 9 h/w
Number (%)	42,362 (67.1)	8,323 (13.2)	13,643 (21.6)	5,394 (8.5)	6,128 (9.7)	4,386 (7.0)
Age, y	56.8 ± 8.0	56.6 ± 7.9	57.9 ± 8.1	53.0 ± 6.7	58.7 ± 8.1	58.2 ± 8.5
Female sex, n (%)	26,482 (62.5)	3,195 (38.4)	8,854 (64.9)	1,802 (33.4)	3,741 (61.1)	2,420 (55.2)
Dialect group, n (%)						
Hokkien	19,296 (45.6)	3,756 (45.1)	5,349 (39.2)	2,606 (48.3)	3,065 (50.0)	2,069 (47.2)
Cantonese	23,066 (54.5)	4,567 (54.9)	8,294 (60.8)	2,788 (51.7)	3,063 (50.0)	2,317 (52.8)
Education, n (%)						
None	13,478 (31.8)	1,678 (20.1)	5,614 (41.2)	463 (8.6)	2,101 (34.3)	1,294 (29.5)
Primary	18,946 (44.7)	3,868 (46.5)	6,057 (44.4)	2,160 (40.1)	2,740 (44.7)	2,074 (47.3)
Secondary school or higher	9,938 (23.5)	2,777 (33.4)	1,972 (14.5)	2,771 (51.4)	1,287 (21.0)	1,018 (23.2)
Smoking, n (%)						
Never	29,919 (70.6)	5,308 (63.8)	9,868 (72.3)	3,402 (63.1)	4,218 (68.8)	2,774 (63.3)
Former	4,150 (9.8)	1,164 (14.0)	1,171 (8.6)	722 (13.4)	734 (12.0)	623 (14.2)
Current	8,293 (19.6)	1,851 (22.2)	2,604 (19.1)	1,270 (23.5)	1,176 (19.2)	989 (22.6)
Hypertension, n (%)	10,115 (23.9)	1,988 (23.9)	2,945 (21.6)	1,281 (23.8)	1,735 (28.3)	1,213 (27.7)
Diabetes, n (%)	3,967 (9.4)	674 (8.1)	1,189 (8.7)	519 (9.6)	674 (11.0)	585 (13.3)
Coronary heart disease, n (%)	1,754 (4.1)	388 (4.7)	534 (3.9)	178 (3.3)	369 (6.0)	263 (6.0)
Stroke, n (%)	695 (1.6)	124 (1.5)	207 (1.5)	73 (1.4)	133 (2.2)	149 (3.4)
Body mass index, kg/m^2^	23.1 ± 3.3	23.2 ± 3.2	23.0 ± 3.1	23.2 ± 3.4	23.1 ± 3.3	23.1 ± 3.5
Body mass index, n (%)						
< 23 kg/m^2^	20,009 (47.2)	4,136 (49.7)	6,167 (45.2)	2,716 (50.4)	2,800 (45.7)	2,136 (48.7)
23–27.5 kg/m^2^	18,617 (44.0)	3,531 (42.4)	6,506 (47.7)	2,162 (40.1)	2,788 (45.5)	1,847 (42.1)
> 27.5 kg/m^2^	3,736 (8.8)	656 (7.9)	970 (7.1)	516 (9.6)	540 (8.8)	403 (9.2)
Physical activity, h/week	-	-	1.40 ± 4.3	1.44 ± 3.7	1.42 ± 4.0	1.36 ± 3.7
Sitting, h/day	6.34 ± 3.6	6.61 ± 3.4	-	-	6.19 ± 3.6	6.41 ± 3.4
Sleep, h/day	7.00 ± 1.2	7.01 ± 1.1	6.94 ± 1.1	7.00 ± 1.1	-	-

The data are expressed as n (%) or mean ± standard deviation.

During a mean follow-up time of 14.5 years (912,764 person-year), 1,973 new cases of TKR were documented. Durations of strenuous sports and vigorous work, either independently or combined, were not associated with risk of TKR. However, moderate activity of 5 or more hours/week was associated with increased risk of TKR when compared to less than 0.5 hour/week in the fully adjusted model (HR 1.16, 95% CI 1.00–1.35). When we summated the duration on all three types of physical activity for each participant, compared to spending less than 0.5 hours/week on physical activity, spending 5 or more hours/week was associated with increased risk of TKR (HR 1.10, 95% CI 0.96–1.26), while spending intermediate duration of 0.5-<2 hours/week was associated with reduced risk (HR 0.88, 95% CI 0.73–1.05), However, we noted that both risk estimates did not reach statistical significance in the fully adjusted model (Model 3) (Table 2). In contrast, there was a significant inverse dose-response relationship between sitting at work and TKR risk (*p* for trend <0.001); participants spending 7 or more hours/day sitting at work were associated with the greatest reduction of in risk compared to those who spent less than 1 hour/day sitting at work (HR 0.63, 95% CI 0.48–0.83) in the fully adjusted Model 3. The association between sitting for TV or other sitting activities, and the risk of TKR was non-conclusive. Total sitting duration was associated with lower risk of TKR in a step-wise manner (*p* for trend = 0.02), with those spending 12 hours or more hours/day sitting having the lowest risk (HR 0.76, 95% CI 0.60–0.96) (Table 3). Finally, we also found a significant inverse association between sleep duration and risk of TKR in a linear fashion (*p* for trend< 0.001); participants with sleeping time ≥ 9 hours/day were associated with the lowest risk compared to those with ≤5 hours/day (HR 0.55, 95% CI 0.43–0.70) in the fully adjusted Model 3 (Table 4).

**Table 2:** Hazard ratio (95% confidence interval) of total knee replacement according to physical activity, the Singapore Chinese Health Study.

	Cases/person-years	Model 1*	Model 2^†^	Model 3^¥^
**Moderate activity**				
< 0.5	1532/708060	1.00	1.00	1.00
0.5-<2 h/week	103/56213	0.92 (0.76–1.13)	0.92 (0.75–1.12)	0.90 (0.74–1.10)
2-<5 h/week	139/72633	0.93 (0.78–1.11)	0.94 (0.79–1.12)	0.93 (0.78–1.11)
≥ 5 h/week	199/75858	1.21 (1.04–1.40)	1.20 (1.04–1.40)	1.16 (1.00–1.35)
*p* for trend^§^		0.11	0.11	0.25
**Strenuous sports**				
No	1901/847611	1.00	1.00	1.00
0.5-<2 h/week	29/29037	0.74 (0.51–1.08)	0.75 (0.52–1.09)	0.73 (0.50–1.05)
2-<5 h/week	27/22044	0.98 (0.67–1.43)	0.99 (0.67–1.45)	0.95 (0.64–1.39)
≥ 5 h/week	16/14072	0.95 (0.58–1.55)	0.94 (0.57–1.54)	0.88 (0.54–1.45)
*p* for trend^§^		0.46	0.48	0.29
**Vigorous work**				
No	1880/843057	1.00	1.00	1.00
0.5-<2 h/week	33/21058	1.22 (0.86–1.73)	1.22 (0.86–1.73)	1.21 (0.86–1.72)
2-<5 h/week	25/16199	1.07 (0.72–1.59)	1.11 (0.75–1.66)	1.14 (0.77–1.70)
≥ 5 h/week	35/32450	1.01 (0.72–1.42)	1.00 (0.71–1.41)	0.99 (0.70–1.39)
*p* for trend^§^		0.67	0.63	0.65
**Total physical activity**				
< 0.5	1413/605931	1.00	1.00	
0.5-<2 h/week	138/87623	0.90 (0.75–1.07)	0.90 (0.75–1.07)	0.88 (0.73–1.05)
2-<5 h/week	170/95829	0.95 (0.81–1.12)	0.96 (0.82–1.13)	0.95 (0.81–1.12)
≥ 5 h/week	252/123381	1.14 (1.00–1.31)	1.14 (1.00–1.31)	1.10 (0.96–1.26)
*p* for trend^§^		0.22	0.20	0.44

^*^ Multivariate model 1: adjusted for age, sex, dialect, year of interview, educational level, smoking status, body mass index, baseline history of self-reported, diabetes, hypertension, coronary heart disease, and stroke

^†^ Multivariate model 2: Model 1 plus further adjustment for sleep (hours/day), sitting duration (hours/day). Specific analysis for strenuous sports, vigorous work and moderate activity also included adjustment for the other two types of physical activity.

^**¥**^ Multivariate model 3: Model 2 plus further adjustment for weekly use of vitamins/minerals, daily energy intake (kcal/day), Alternate Healthy Eating Index (AHEI) score, menopausal status and use of hormonal replacement therapy in women

^§^ Linear trend was tested by treating the categories as a continuous variable.

**Table 3:** Hazard ratio (95% confidence interval) of total knee replacement according to physical activity, the Singapore Chinese Health Study.

	Cases/person-years	Model 1*	Model 2^†^	Model 3^¥^
**Sitting at work**				
<1 h/day	1623/651185	1.00	1.00	1.00
1-<3 h/day	129/78886	0.80 (0.67–0.96)	0.80 (0.67–0.96)	0.79 (0.66–0.94)
3-<7 h/day	163/120481	0.84 (0.71–1.00)	0.85 (0.72–1.00)	0.84 (0.71–1.00)
≥ 7 h/day	58/62212	0.62 (0.48–0.82)	0.63 (0.48–0.82)	0.63 (0.48–0.83)
*p* for trend^§^		<0.001	<0.001	<0.001
**Sitting for TV**				
<1 h/day	268/159593	1.00	1.00	1.00
1-<3 h/day	812/404318	1.07 (0.93–1.23)	1.09 (0.95–1.26)	1.07 (0.93–1.23)
3-<5 h/day	774/296181	1.16 (1.01–1.33)	1.19 (1.03–1.36)	1.17 (1.01–1.34)
≥ 5 h/day	119/52672	0.96 (0.77–1.20)	0.96 (0.77–1.19)	0.94 (0.75–1.17)
*p* for trend^§^		0.26	0.23	0.32
**Other sitting activities**				
<1 h/day	1156/2467656	1.00	1.00	1.00
1-<3 h/day	576/318622	0.96 (0.86–1.07)	0.97 (0.87–1.08)	0.96 (0.86–1.07)
3-<5 h/day	170/99674	0.80 (0.68–0.95)	0.80 (0.68–0.95)	0.81 (0.68–0.95)
≥ 5 h/day	71/26813	1.14 (0.89–1.45)	1.11 (0.87–1.41)	1.11 (0.87–1.42)
*p for trend§*		0.25	0.21	0.23
**Total sitting activities**				
<4 h/day	511/198446	1.00	1.00	1.00
4-<6 h/day	598/243862	1.00 (0.89–1.13)	1.01 (0.90–1.14)	1.00 (0.89–1.13)
6-<8 h/day	468/201152	1.02 (0.90–1.16)	1.04 (0.91–1.18)	1.03 (0.91–1.18)
8-<12 h/day	309/189742	0.86 (0.74–1.00)	0.87 (0.75–1.01)	0.87 (0.75–1.00)
≥ 12 h/day	87/79563	0.76 (0.60–0.96)	0.76 (0.60–0.97)	0.76 (0.60–0.96)
*p* for trend^§^		0.01	0.02	0.02

* Multivariate model 1: adjusted for age, sex, dialect, year of interview, educational level, smoking status, body mass index, baseline history of self-reported, diabetes, hypertension, coronary heart disease, and stroke

^†^ Multivariate model 2: further adjusted for duration of sleep (hours/day) and total physical activity (hours/week). Specific analysis for sitting at work, sitting for TV and other sitting activities also included adjustment for the other two types of sitting activities.

^**¥**^ Multivariate model 3: further adjusted for weekly use of vitamins/minerals, daily energy intake (kcal/day), Alternate Healthy Eating Index (AHEI) score, menopausal status and use of hormonal replacement therapy in women

^§^ Linear trend was tested by treating the categories as a continuous variable.

**Table 4:** Hazard ratio (95% confidence interval) of total knee replacement according to sleep duration, the Singapore Chinese Health Study.

	Cases/person-years	Model 1*	Model 2^†^	Model 3^¥^
**Sleep duration**				
≤ 5 h/day	246/83809	1.00	1.00	1.00
6 h/day	519/212259	0.91 (0.78–1.06)	0.91 (0.78–1.06)	0.91 (0.78–1.06)
7 h/day	646/304560	0.81 (0.70–0.94)	0.82 (0.70–0.95)	0.82 (0.70–0.95)
8 h/day	471/252585	0.72 (0.62–0.85)	0.73 (0.62–0.85)	0.74 (0.63–0.86)
≥ 9 h/day	91/59551	0.54 (0.42–0.69)	0.54 (0.43–0.69)	0.55 (0.43–0.70)
*p* for trend^§^		<0.001	<0.001	<0.001

^*^ Multivariate model 1: adjusted for age, sex, dialect, year of interview, educational level, smoking status, body mass index, baseline history of self-reported, diabetes, hypertension, coronary heart disease, and stroke

^†^ Multivariate model 2: Model 1 plus further adjustment for total physical activity (hours/week) and total sitting duration (hours/day).

^**¥**^ Multivariate model 3: Model 2 plus further adjustment for weekly use of vitamins/minerals, daily energy intake (kcal/day), Alternate Healthy Eating Index (AHEI) score, menopausal status and use of hormonal replacement therapy in women

^§^ Linear trend was tested by treating the categories as a continuous variable.

In the sensitivity analysis that excluded 10,260 participants, including 320 cases, that had imputed BMI values due to missing information on weight or height, the results remained essentially the same for the positive association between 5 or more hours/week of moderate activity and TKR risk, as well as for the inverse relationship of sitting and sleep duration with TKR risk (data not shown). There was no significant interaction between BMI categories (<23kg/m^2^ and ≥23kg/m^2^) and duration of moderate physical activity, sitting or sleep for TKR risk (all *p* values for interaction > 0.16). For the interaction with gender, the inverse association between hours of sitting and TKR risk was stronger in men compared to women (*p* for interaction = 0.04); compared to those who sat for less than 4 hours/day, the HR (95% CI) for those who sat for 12 or more hours/day was 0.44 (0.28–0.71) in men (*p* for trend<0.001), and 0.95 (0.72–1.25) in women (*p* for trend = 0.36), in the fully adjusted Model 3. Again, this was mainly due to the much strong inverse association between hours of sitting at work and TKR risk in men compared to women (*p* for interaction = 0.005); compared to those who sat for <1 hour/day at work, the HR (95% CI) for those who sat for 7 or more hours/day was 0.45 (0.28–0.72) in men (*p* for trend<0.001), and 0.73 (0.53–1.01) in women (*p* for trend = 0.04) in the fully adjusted Model 3. There was no interaction between gender and hours of moderate physical activity or sleep for TKR risk (all *p* values for interaction>0.41).

Since we asked our participants about duration of physical activity in hours per week, and the durations for sitting and sleeping in hours per day, when we summated the hours of physical activity, sleep and sitting, there were 1,012 participants (1.6%), including 9 cases, who exceeded 24 hours a day. We repeated all analyses by excluding these 1,012 participant, and the results are virtually identical. Finally we conducted sensitivity analysis by excluding those with less than 5 years of follow-up, and all the findings for the associations of TKR risk with durations of moderate physical activity, sitting and sleep remained materially unchanged.

## Discussion

Generally, this prospective population-based cohort encompassed a relatively non-active population with a sedentary urban city lifestyle, which is comparable to other developed countries that are also ageing [19–20]. In this population, while increasing duration of sitting and sleeping, which are non-weight bearing activities, could be associated with reduced risk of having to undergo TKR for severe KOA, excessive moderate physical activity of 5 or more hours per week could be associated with increased risk.

There is substantive evidence in support of the benefits of structured prescribed therapeutic exercises programs (both land-based and water-based) in reducing knee pain and improving physical function for people with KOA [4, 21], and exercise plays central role in the management of KOA [3, 22]. However, physical activity is defined as ‘‘any bodily movement produced by skeletal muscles that results in energy expenditure”, and is a broader concept than exercise as it comprises all occupational, transport, domestic, and leisure time activities [23]. We previously demonstrated the log-linear relationship between BMI and risk of TKR due to severe KOA across a wide range of BMI, and provided evidence that biomechanical loading on the knee joints plays a pivotal role in KOA initiation and/or progression [2]. Indeed, individuals who frequently have high weight loading on their knee joints from sports, occupational activities, and squatting have been shown to experience higher risk of KOA [24–26]. Hence, while physical activity can strengthen the muscles supporting the knee, reduce the risk of joint injuries, and prevent obesity, it can also increase weight-loading on the knees. It is therefore not unexpected that the findings on the association of day-to-day physical activity with onset or progression of KOA in the general population in epidemiologic studies have been conflicting.

Among the prospective cohort studies with long follow-ups, two [27–28] have demonstrated a protective role against disability in KOA, five [29–33] did not show any association, while others [8, 26, 34–36] demonstrated physical activity as a risk factor to onset or progression of KOA. A systematic review on ten high quality prospective studies concluded that no consensus can be drawn regarding the effect of physical activity on development of KOA due to inconsistent findings [4]. Apart from differences in study design and characteristics of target populations, variability in the intensity, duration, and nature of physical activity was the main reason for the conflicting results in epidemiological studies. A recent meta-analysis evaluated the association of recreational and competitive running with KOA, and demonstrated a U-shaped relationship: recreational runners had a lower occurrence of KOA compared with competitive runners and sedentary non-running controls [6]. Conversely, some cohort studies revealed increased risk of incident KOA only at high levels of physical activity [34–36]. This is consistent with our finding that the risk of TKR due to severe KOA was only seen in those with 5 or more hours per week of moderate physical activity, while those with shorter duration had slight reduction in risk that did not reach statistical significance. In this population, small numbers of participants were engaged in strenuous activity or vigorous work, and we acknowledge that this study is underpowered to examine the effect of these activities on TKR risk.

Apart from the intensity of physical activity, we also postulate that the type of physical activity is important. In a large prospective population study involving 315,495 participants and 12-year of follow-up, Apold *et al*. demonstrated that physical activity at work, rather than physical activity at leisure, had a dose-dependent relationship with risk of TKR [26]. However, most physical activity questionnaires used in epidemiological studies concentrate on intensity and frequencies, and lack the differentiation of types, particularly between weight-bearing and non-weight bearing activities [37]. Different types of physical activity may have different effect to weight-bearing joints. Among college athletes undergoing high-intensity training, biomarkers of bone resorption and cartilage collagen degradation (NTXI and CTXII) were higher among crew rowers and runners than in swimmers or controls, after adjustment for BMI [38]. A study that used gait analysis for knee loading has found that the rate and magnitude of knee adduction moment loading during walking were independent factors associated with degenerative changes of the knee joint on MRI [39]. Other clinical studies have attempted to measure weight-loading and physical activity using objective measures. In the Osteoarthritis Initiative Study, pedometers were utilized to provide more accurate measurement of walking activities, and in this cross-sectional study, participants with meniscal and bone marrow edema lesions on MRI had higher average of physical activity in minutes per day compared to those without these lesions [7]. In a prospective cohort followed up for over 2.7 years, Dore et al. demonstrated that pedometer-tracked physical activity ≥10,000 steps per day increased risk of increasing meniscal pathology, bone marrow edema lesions, and cartilage defects, particularly for subjects with worse defects at baseline [8]. At moderate intensity, it is estimated to take about 100 minutes to complete 10,000 steps [40]. Hence, these studies corroborated with our finding of an increased risk of TKR in participants with at least 5 hours of physical activity, most of which was moderate activity that involved brisk walking.

On the other hand, our study showed an inverse association between sitting at work and the risk of TKR in a dose-dependent manner, which is generally consistent with other studies that have reported a protective role of sitting in relation to KOA [41–43]. Specifically, a dose-dependent relationship between cumulative hours of occupational related sitting and KOA was also reported in a case-control study [43]. We are not surprised that the association with sitting for TV or other sitting activities was non-conclusive because from our interaction with our participants, we realised that unlike the certainty in reporting hours of sitting at work, the participants were less certain of their hours spent watching TV or in other sitting activities as the activities occurred intermittently throughout the day. Hence, these two sitting activities could have more misclassification errors than sitting at work. In our study, the inverse association between sitting at work and TKR risk was stronger in men than in women, and we postulate that this could reflect the difference in the nature of work that required standing in both genders. It is possible that men who did not sit at work could be involved in more vigorous and physically demanding tasks that had more weight-bearing effect on the knees, compared to women who did not sit at work. Future studies would be needed to validate this finding.

It was not surprising that we also found a stepwise reduction in risk of TKR with prolonged sleep duration. While previous studies have focused on poor sleep quality as a result of the pain and other symptoms of KOA, and the improvement in sleeping time and quality after joint replacement surgeries, no study has evaluated the duration of sleep in relation to risk or progression of KOA. We postulate that during sitting or sleeping, the “unloading” of weight on the knee joints could facilitate cartilage repair, a process analogous to the effects of mechanical unloading in joint distraction surgeries.

The strengths of our study included a large number of TKR cases identified from a prospective population-based cohort with long follow-up time. The case ascertainment of TKR through linkage with the comprehensive, nationwide hospital database could be considered complete. We included both established risk factors for severe KOA and other possible risk factors as covariates in our analyses, and the collection of information on exposure and covariate factors prior to TKR minimized recall bias. However, the study has to be interpreted in light of its limitations. We used TKR as a surrogate for severe KOA, and we cannot discern the association with exposures were related to onset or progression of KOA. We evaluated physical activity using a modified version of the validated EPIC physical activity questionnaire [10] and did not include any objective assessment. Nevertheless, the self-reported duration of physical activity in this Singapore Chinese Health Study cohort has been shown to be associated with risk of diseases such as cardiovascular disease mortality and end-stage kidney disease [44–45]. We only used one-time point assessment of physical activity and thus could not determine the relationship between lifetime cumulative physical activity level or changes in physical activity over time, and the risk of TKR. Our results should be interpreted with caution since reverse causation or temporal bias could not be excluded. For example, participants with chronic knee pain from severe KOA might have changed their physical activity level to become less physical activity and spend longer hours in sitting activities or sleeping to cope with their symptoms, and thus avoid the need for TKR. This could therefore lead to an underestimation of the risk estimates. To minimize this, we conducted sensitivity analysis by excluding those with less than 5 years of follow-up, and all the findings for the associations of TKR risk with durations of physical activity, sitting, and sleep remained materially unchanged. We did not collect information of specific type of supplements use like glucosamine or chondroitin. However, evidence supportive of their roles in slowing down OA progression has not been unequivocally established. Finally, we did not collect data on knee injury, which is a known confounder to physical activity as a risk factor for KOA. We also did not collect detailed information about work to ascertain the extent of knee-bearing activities for those who did not sit at work.

In conclusion, this study provided evidence that while increased non-weight bearing activities such as sitting and sleeping could be associated with reduced risk of severe KOA, prolonged physical activity of 5 hours or more per week, especially of the weight-bearing type, could have detrimental effect. While we do not advocate prolonged sitting and sleeping as a means of preventing onset or progression of KOA, our observations have practical implications in understanding the benefit of reducing excessing loading of weight on the knee joints as a means to slow down progression of KOA.
