# Correction: Duration of physical activity, sitting, sleep and the risk of total knee replacement among Chinese in Singapore, the Singapore Chinese Health Study

**DOI:** 10.1371/journal.pone.0205547

**Published:** 2018-10-05

**Authors:** Ying Ying Leung, Hamid Rahmatullah Bin Abd Razak, Mohammad Talaei, Li-Wei Ang, Jian-Min Yuan, Woon-Puay Koh

The second author’s name appears incorrectly in the citation. The author’s first name is Hamid Rahmatullah. The author’s last name is Bin Abd Razak. The correct citation is: Leung YY, Bin Abd Razak HR, Talaei M, Ang L-W, Yuan J-M, Koh W-P (2018) Duration of physical activity, sitting, sleep and the risk of total knee replacement among Chinese in Singapore, the Singapore Chinese Health Study. PLoS ONE 13(9): e0202554. https://doi.org/10.1371/journal.pone.0202554

## References

[1] Leung YY, Razak HRBA, Talaei M, Ang L-W, Yuan J-M, Koh W-P (2018) Duration of physical activity, sitting, sleep and the risk of total knee replacement among Chinese in Singapore, the Singapore Chinese Health Study. PLoS ONE 13(9): e0202554 10.1371/journal.pone.0202554 30180156 PMC6122790

